# Maximum Power Game as a Physical and Social Extension of Classical Games

**DOI:** 10.1038/srep43649

**Published:** 2017-03-08

**Authors:** Pilwon Kim

**Affiliations:** 1 Ulsan National Institute of Science and Technology(UNIST) Department of Mathematical Sciences, Ulsan Metropolitan City, 689-798, South Korea

## Abstract

We consider an electric circuit in which the players participate as resistors and adjust their resistance in pursuit of individual maximum power. The maximum power game(MPG) becomes very complicated in a circuit which is indecomposable into serial/parallel components, yielding a nontrivial power distribution at equilibrium. Depending on the circuit topology, MPG covers a wide range of phenomena: from a social dilemma in which the whole group loses to a well-coordinated situation in which the individual pursuit of power promotes the collective outcomes. We also investigate a situation where each player in the circuit has an intrinsic heat waste. Interestingly, it is this individual inefficiency which can keep them from the collective failure in power generation. When coping with an efficient opponent with small intrinsic resistance, a rather inefficient player gets more power than efficient one. A circuit with multiple voltage inputs forms the network-based maximum power game. One of our major interests is to figure out, in what kind of the networks the pursuit for private power leads to greater total power. It turns out that the circuits with the scale-free structure is one of the good candidates which generates as much power as close to the possible maximum total.

Imagine a situation where an adjustable resistor is placed between two wires connected to distinct voltage sources and you want to draw the maximum power generation on it. As in Fig. 1(a), if the fixed resistance of the wires is *R*_*c*_, then you can obtain the maximum power out of the resistor by setting its resistance to *r* = 2*R*_*c*_, according to the maximum power transform theorem^1^. The corresponding maximum power output is *V*^2^/8*R*_*c*_ where *V* = *V*_2_ − *V*_1_.

Now let us further consider a game of two agents *r*_1_ and *r*_2_ in the parallel circuit as in Fig. 1(b). Whenever an agent checks its current power, it finds that its power tends to increase each time it lowers its resistance. The problem is, however, that this choice sharply reduces the other side’s power production. The agents keep lowering their resistance until both of them eventually lose all resistance. This implies that neither of them get to generate any power in the end.

What if the circuit is more complicated with multiple resistors and each of them is trying to get its own maximum power? Fig. 1(c,d) show two examples of such cases. In these circuits, the current flowing through one resistor is influenced not only by its resistance, but also by the resistance of others in a complex way. So they react simultaneously to the current they experience, and control their resistance to raise their power. This turns out to be a continuous multi-player game.

This, the maximum power game, is the evolutionary game with continuous strategy space. It is close to replicator dynamics which has been studied in economics, population biology, and machine learning^2^,^3^,^4^ in that their dynamics are naturally described in evolutionary differential equations. However, while replicator dynamics mostly describes the evolution of the population density across finite traits, we trace the evolution of a continuous trait(resistance) of the finite agents in the maximum power game.

In this work, we focus on theoretical aspect of the maximum power game, as a physical/social extension of classical games. Summation of the individual powers at equilibrium is generally not the maximum power that the system can generate. Indeed, the individual efforts to increase their own power often end up at the worst possible Nash equilibrium - no power production at all. This degeneracy implies similarity between the maximum power game and the prisoner’s dilemma game. One can also find a similar aspect in social dilemmas involving resource depletion that have long been studied in economics^5^,^6^. However, the maximum power game is not a simple physical analogy of the prisoner’s dilemma game. In the following sections, we will see the opposite situation is often created with a certain condition, in which the individual pursuit of maximum power promotes the decent collective outcomes.

Within the context of power control problem, the game-theoretical framework have been used to study several applications in the design and analysis of reliable and efficient electrical power systems^7^,^8^,^9^. If we further assume that the agents have memory and their decisions depend on the history, the resistors can be regarded as memristors. Memristors are passive components that behave as resistors with memory. Memristive systems show the various complex behavior including hyperchaos, scale invariance, and time nonlocality^10^,^11^,^12^. However, the agents implemented in this work are simple resistors that refer only to their current power. The purpose of this paper is to present the most concise form of physical modules that can be extended to contain social implications and has a common denominator with the existing game theory.

This work studies the unique features of the maximum power games, focusing on two factors that affect the resource distribution at equilibrium: circuit topology and player efficiency. In the maximum power game, all players’ choices are organically connected and are sensitively influenced by one another immediately, even when they are located far away in the circuit. Another important point is the relation between the efficiency of the players and the efficiency of the whole system. If the players are not an ideal power generator and inevitably produce some heat waste, which is true in real world, their game may result in a completely different distribution even in a simple circuit.

System’s pursuit for the maximum power has been proposed as a formal principle in open system thermodynamics and ecology. According to ref. 13, “During self-organization, system designs develop and prevail that maximize power intake, energy transformation, and those uses that reinforce production and efficiency”. As mentioned above, competition over a simple structure likely leads to catastrophic failure in the whole system. Hence the question here is: under what kind of structure, the subsystems’ pursuit for the maximum power consistently leads to the maximum power at larger scales? If natural selection works as a maximum power organizer, such structure that generates the maximum power consistently across scales is selected. In this work, we show the corresponding system is the circuits with the scale-free structure.

## Dynamics of the Maximum Power Games

Dynamics of the game can be understood as the continuous limit of the following repeated discrete game. The players change their resistances, *r*_1_, *r*_2_, 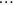, *r*_*N*_, at *t*_*k*+1_ = *t*_*k*_ + Δ*t, k* = 1, 2, 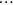. The *i*th player checks its own power *w*_*i*_ = *w*_*i*_(*r*_1_, *r*_2_, 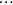, *r*_*N*_) at the moment and decides whether to raise or lower *r*_*i*_. Each player has no information on other parts of the circuit, except its own current power as a feedback from the system. The process can be modelled as


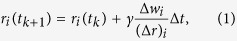


where 0 < Δ*r* ≪ 1,Δ*t* and *γ* > 0.

As Δ*t* and Δ*r* → 0, we obtain


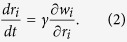


We say the above system reaches a Nash equilibrium when if no player attempts to change its resistance. This implies, if 
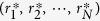
 satisfies


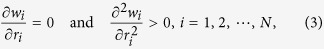


it is a Nash equilibrium.

Once *w*_*i*_, *i* = 1, 2, 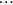, *N* is found as a function of the resistances from the circuit topology, one can analyze the systems behaviour from the differential equations (2). All the results of this paper are obtained in such manner. However, for the network-based maximum power games in the last section, we used the Monte Carlo simulations based on the discrete scheme (1) due to extreme complexity of ∂*w*_*i*_/∂*r*_*i*_.

**Figure 1:**
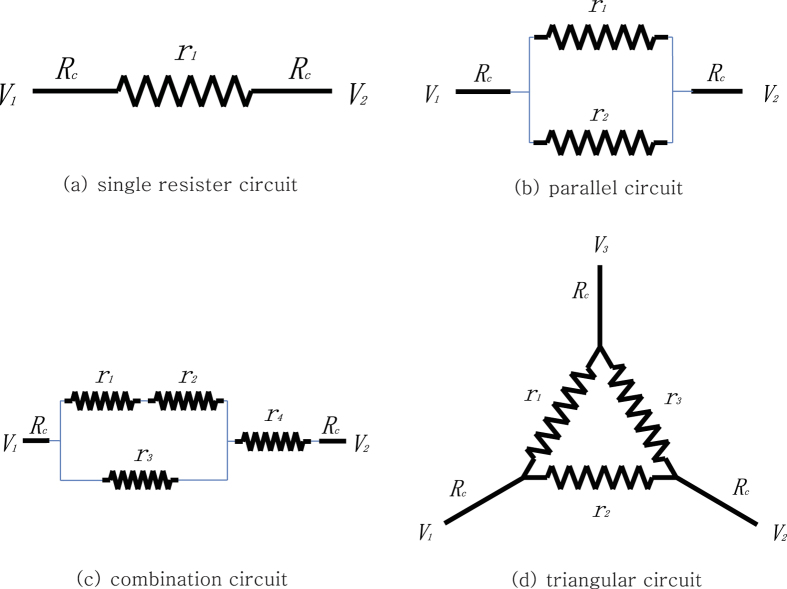
Circuits with varying resistors.

As a concrete example of the dynamics, let us take a parallel game in Fig. 1(b). The voltages and the currents on the parts of the circuit follow the systems of equations as


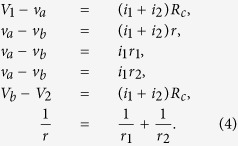


Here *v*_*a*_ and *v*_*b*_ denote the voltage at the left and the right branching point, respectively, and *i*_1_ and *i*_2_ denote the current flowing through *r*_1_ and *r*_2_, respectively. Solving the above equations for those variables with respect to *r*_1_ and *r*_2_, one can express the powers and as functions of *r*_1_ and *r*_2_ as


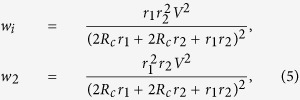


The dynamics of the game is now obtained from (2) as


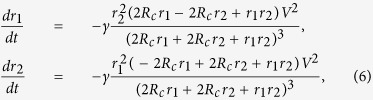


**Figure 2:**
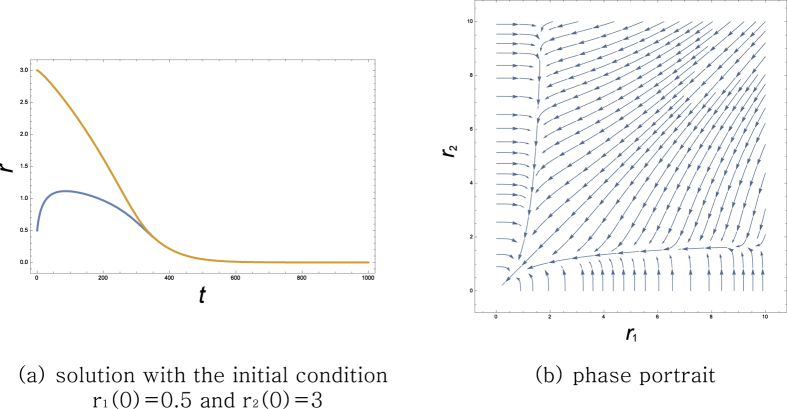
2-player parallel game.

The phase portrait and a sample solution of the system are shown in Fig. 2. The only Nash equilibrium of the system is 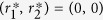.

## Equilibriums in Simple Maximum Power Games

**Figure 3:**
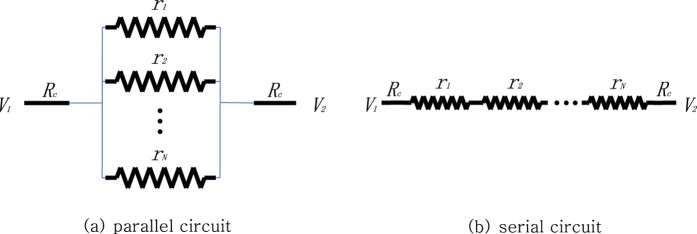
Basic circuits with *N* varying resistances.

In this section, we study the maximum power game in the basic circuits with a single potential difference. Figure 3 illustrates examples of parallel and serial circuits lying between one potential difference. We assume that all agents are ideally efficient and can adjust their resistivity from 0 to infinity, making themselves a superconductor to a perfect insulator, respectively.

### Parallel games

Let *V* = *V*_2_ − *V*_1_ denote voltage difference and let *R*_*c*_ be a constant resistance of the connecting wires as in Fig. 3(a). Let *r*_1_, *r*_2_, 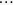, *r*_*N*_ be the resistance of the *N* parallelly-placed resistors. The power generated at the *i*-th resistor is evaluated as


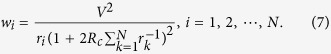


Since the agent is trying to maximize its power by adjusting the resistance *r*_*i*_, the equilibrium can be found from the equations


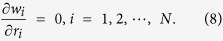


which gives 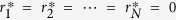 and therefore *w*_1_ = *w*_2_ = 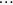 = *w*_*N*_ = 0. This implies that the agents turn their resistivity down competitively to raise their power and eventually end up in the worst situation in which no player benefits from resource.

### Serial games

Consider the *N* agents serially connected as in Fig. 3(b). This time the power of *i*-th agent is


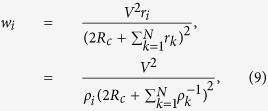


where *ρ*_*i*_ = 1/*r*_*i*_. Solving 
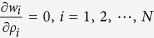
 gives 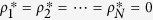 or 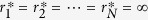. This time all agents make themselves an insulator in the end, which ironically leads to the same result in terms of power generation, i.e., *w*_1_ = *w*_2_ = 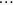 = *w*_*n*_ = 0.

**Figure 4:**
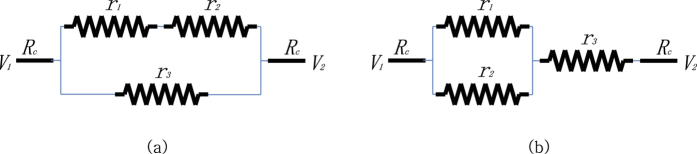
Combination circuits: Equilibriums are (**a**) 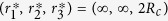 (**b**) 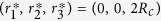.

It can be further shown that any simple composite of parallel/serial circuits leads to a trivial result. Figure 4 presents two examples of circuits that combine parallel/serial components. The resistors *r*_1_ and *r*_2_ in (a) are serially connected and therefore must be infinity at equilibrium, since otherwise they always have incentive to raise their resistance further. This implies the above wire is broken and the circuit becomes a single resistor circuit with *r*_3_ = 2*R*_*c*_ at equilibrium. Similarly, the parallel resistors *r*_1_ and *r*_2_ in (b) must be 0 at equilibrium. Otherwise the status cannot be an equilibrium since they have incentive to lower their resistance further. In this manner, for any combination of parallel/serial elements, one can determine the resistance at equilibrium one by one and eventually simplify the given circuit into trivial one.

When played by ideally efficient agents on parallel/serial circuits or their combinations, the maximum power game results in trivial power distribution. So one of the possible ways to avoid these no-win situations is playing the game in nonstandard circuits.

### Games on Irregular Composite Circuits

**Figure 5:**
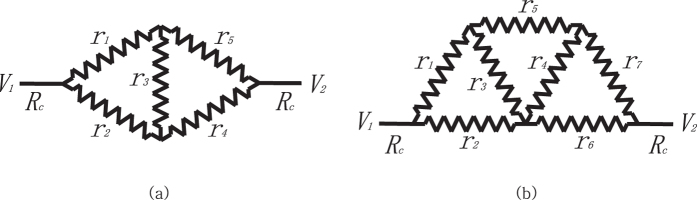
Nonstandard circuits.

Now let us consider the systems with a nonstandard topology. Figure 5 shows some circuits which are not decomposable to a combinations of serial/parallel connections. It turns out that the maximum power game on these systems leads to a nontrivial power distribution. One can show that the circuit in Fig. 5(a) follows the systems of equations as


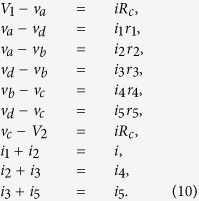


Here *i* denotes the current flowing through *R*_*c*_ and *i*_*k*_, *k* = 1, 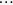, 5 denotes the current flowing through *r*_*k*_. Also, *v*_*a*_, *v*_*b*_, *v*_*c*_ and *v*_*d*_ are the voltages at the nodes, assigned counterclockwise from the left end of *r*_1_. Solving the above equations for these variables with respect to *r*_*k*_, one can express the powers 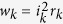 as a function of *r*_1_, 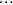, *r*_5_. Then application of the condition (3) gives infinitely many equilibriums as


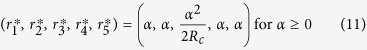


with the corresponding power


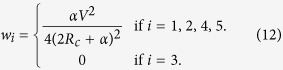


Note that the circuit in Fig. 5(a), without the middle component *r*_3_, would become a simple combination of parallel/serial circuits and therefore results in zero equilibrium. Interestingly, even though the presence of *r*_3_ is essential not to make trivial equilibrium, *w*_3_ remains all the way zero. So the collective failure cannot be avoided without the sacrifice of 3rd agent.

## Agents with Intrinsic Heat Dissipation

Even though the above examples show the characteristic features of the maximum power game, they are unrealistic in that each agent is an ideal resistor which can adjust its resistivity from 0 to infinity.

**Figure 6:**
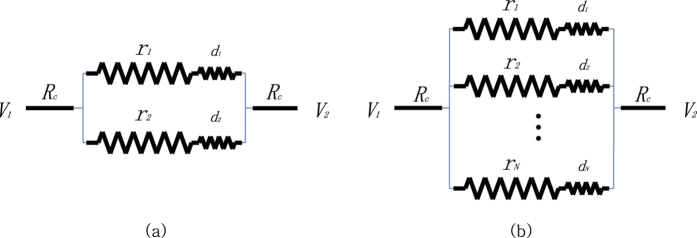
Circuits with agents that have extra dissipative resistance *d*.

In this section, we assume that all agents have internal intrinsic heat waste. The agents have no control over a fixed dissipative resistance, say *d*, and this causes unavoidable heat loss which cannot be converted to usable power. So the agents are trying to maximize their power anywhere but this fixed resistance. The agent with larger *d* tends to lose more heat and therefore 1/*d* can be interpreted as an indicator for the agent’s efficiency. Figure 6 illustrates parallel games with the agents who have a serially connected dissipative resistance.

Suppose as in Fig. 6(a) that two agents with adjustable resistance *r*_1_ and *r*_2_ and with fixed dissipative resistance *d*_1_ and *d*_2_, respectively, are playing the maximum power game in the parallel circuit. Derivation of the equilibrium on this circuit is the same as the 2 player parallel game described in Section 2, except that the equation 1/*r* = 1/*r*_1_ + 1/*r*_2_ in the equations (4) is replaced by 1/*r* = 1/(*r*_1_ + *d*_1_) + 1/(*r*_2_ + *d*_2_). The Nash equilibrium 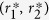 is found as


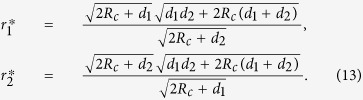


**Figure 7:**
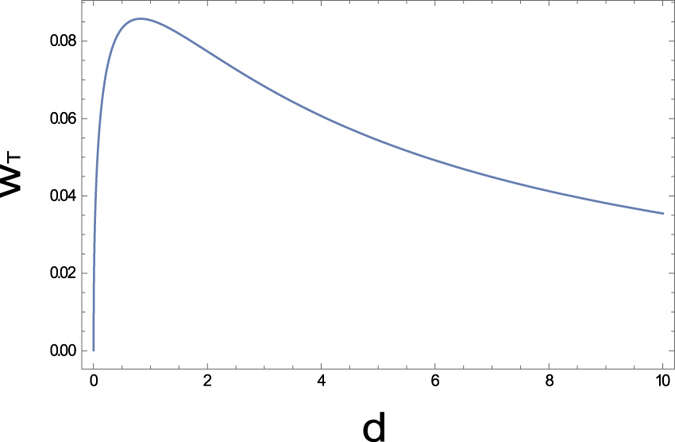
Grpah of the total power *w*_*T*_ = *w*_1_ + *w*_2_ at equilibrium according to the dissipative resistance *d* = *d*_1_ = *d*_2_ in Figure. The total power becomes zero as *d* approaches either 0 or ∞. The parameters *V* = 1 and *R*_*c*_ = 1 are used.

The graph in Fig. 7 shows the total power *w*_*T*_ = *w*_1_ + *w*_2_ generated by two agents at the equilibrium, when the intrinsic resistances are identical as *d*_1_ = *d*_2_ = *d*. One can see that collective power failure in Section 2 can be avoided, ironically from the fact that the agents are not as efficient as they can control their resistivity completely.

**Figure 8:**
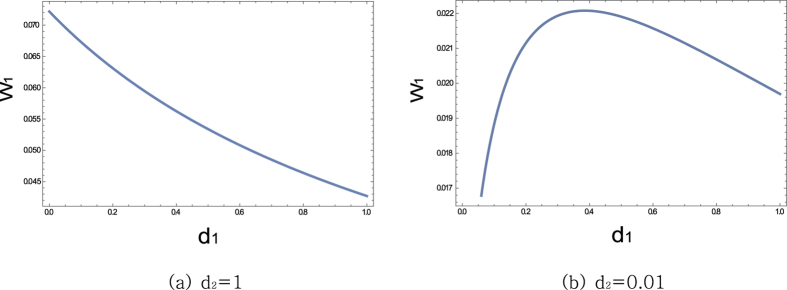
Graphs of the power *w*_1_ according to the dissipative resistance *d*_1_, with the opponent’s dissipative resistance *d*_2_ fixed in Fig. 6(a). When the opponent is efficient with *d*_2_ low at 0.01, the maximum power of *w*_1_ is attained with *d*_1_ at a rather large value around 0.4. The parameters *V* = 1 and *R*_*c*_ = 1 are used.

Now let us investigate the case of *d*_1_ ≠ *d*_2_, where the efficiency of agents are distinct. Figure 8(a) shows the change of the first agent’s power according to its intrinsic resistance level *d*_1_, while the *d*_2_ of the second player is fixed at a large value. One can see that, if the opponent is inefficient with large heat waste, then the agent with smaller *d*_1_ achieves more power. This agrees well with the common sense that an efficient agent beats inefficient ones.

On the contrary, if the opponent is highly efficient with small intrinsic waste *d*_2_, then the first agent’s being more efficient is no more beneficial in the maximum power game. In Section 2, we saw the multiple agents with ideal efficiency end up in the no-win situation. Figure 8(b) shows the power from *r*_1_ according to *d*_1_, with *d*_2_ fixed at 0.01. The best agent who can get the possible maximum power is not the one with small *d*_1_, but the one with a relatively large *d*_1_ around 0.4. This implies that, if one player is highly efficient, the better partner is an inefficient one. Two competing efficient players do not prosper together.

**Figure 9:**
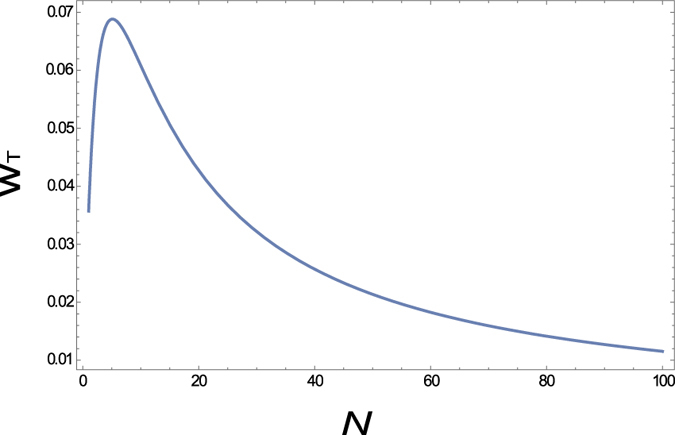
The graph of the total power *w*_*T*_ according to the number of agents *N* in Fig. 6(b). The parameters *V* = 1, *R*_*c*_ = 1 and *d*_1_ = *d*_2_ = 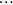 = *d*_*N*_ = 1 are used.

Let us now consider the collective efficiency of multiple agents with heat waste. If you employ *N* homogeneous agents in the parallel circuit and try to harvest as much total power as possible through the maximum power game, how many agents are optimal for the maximum total? If the homogeneous dissipative resistance *d* is assumed, one can find the corresponding total power from the maximum power game as


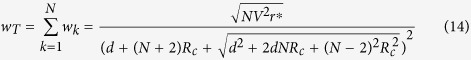


where 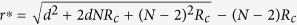 is the corresponding value of the resistances *r*_1_, *r*_2_, 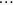, *r*_*N*_ at equilibrium. The example in Fig. 9 shows the optimal number of agents to produce the maximum total power is 7 when *d* = 5, *V*_1_ = 0, *V*_2_ = 1, and *R*_*c*_ = 1. Employing more agents gradually decreases the total power.

For a large value of *d*, the optimal number of the agents N^*^ for the maximum total power is approximately *d*/*R*_*c*_. More precisely,


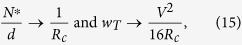


as *d* → ∞.

Note that, no matter what *d* and *N* are, the corresponding total power from the maximum power game cannot exceed the half of the possible maximum power, *V*^2^/8*R*_*c*_. This observation naturally brings us to the next important question: how can we improve the collective efficiency through the maximum power game? If not a simple parallel/serial structure, under what structure can the collective power be promoted by the selfish individuals through the game? In the next section, we investigate this problem, given with the network with multiple voltage sources.

## Maximum Power Games with Multiple Potentials

One of the intriguing challenges in social science is to find a social structure that can reconcile the individual’s pursuit of private interest with improvement of the common good. In this section, we try to study this problem in the context of the maximum power game, by investigating the relation between the circuit topology and the induced total power.

The results of the network-based game discussed in this section are all derived from the numerical experiments performed on the network ensemble. Once the power functions *w*_*i*_ = *w*(*r*_1_, *r*_2_, 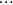, *r*_*N*_), *i* = 1, 2, 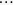, *N* are determined from a given network topology, one can, in principle, describe its dynamics and equilibriums, from (2) and (3). However, this is often too complicated even for small-size networks. (If the networks are a bit larger, finding *w*_*i*_ in the analytic form becomes challenging as well).

Here we apply the Monte Carlo simulation to the scheme (2) in order to search equilibriums of the system; At every time *t*_*k*_, *k* = 1, 2, 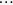, we select an arbitrary resistor and give a random perturbation to its resistance. If the change brings its power up, we assume that the agent chooses the change of resistance, otherwise remains the same. We repeat this process until the system reaches the stage where no more agents are trying to change their resistance. Note that having all players choose in turn (sequential updating), or a randomly selected player choose(random updating), or everyone choose all at once(synchronous updating), do not make substantive difference in the results.

In all the examples, we have created 1000 networks of the same nature with 30 nodes. For example, in the case of the Erdős-Rnyi network, 1000 networks were created and tested for every *p* value. We performed the Markov Chain Monte Carlo(MCMC) to prevent the outcomes to be stuck in a local minimum. The results were compared in the mean and the standard deviation.

**Figure 10:**
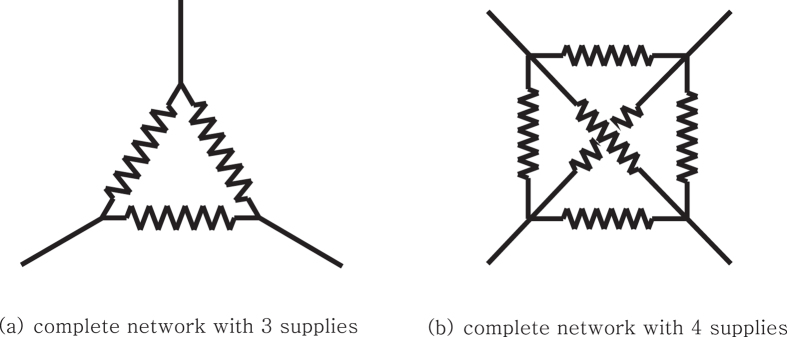
Network circuits with multiple potentials. 3 agents and 6 agents are placed in the complete networks in (**a**,**b**), respectively.

Let us consider a circuit with multiple external voltages *V*_1_, *V*_2_, 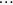, *V*_*N*_. Each external voltage is supplied through a wire with a fixed resistance *R*_*c*_. To simplify the setting, we assume the each agent directly connects two of the voltage-supplying wires, from *V*_*i*_ and *V*_*j*_, and is denoted by *r*_*ij*_. We also assume there is no heat drain from the agents. In the light of the network theory, the agents can be regarded as links connecting the supplying nodes (the end of supplying wires). Figure 10(a and b) shows two examples of such network circuits which have complete connections with 3 and 4 supplies, respectively.

The maximum power that can be generated from the complete network circuit is


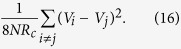


This is not analytically proved yet but it has been tested through numerical experiments with complete networks of various size. Note that this maximum is generally not attainable from the maximum power game, but from the careful coordination of *r*_*ij*_. More precisely, it is observed that the possible maximum power is generated if all agents’ resistances *r*_*ij*_ follow the relation


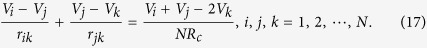


It is however not possible to satisfy the above equations through the maximum power game. (One of the simple solutions to the equations is *r*_*ij*_ = *NR*_*c*_, but it is not a stable Nash equilibrium of the game). In fact, the maximum power game on any complete connected networks with *N* > 3 external supplies results in trivial zero equilibrium.

**Figure 11:**
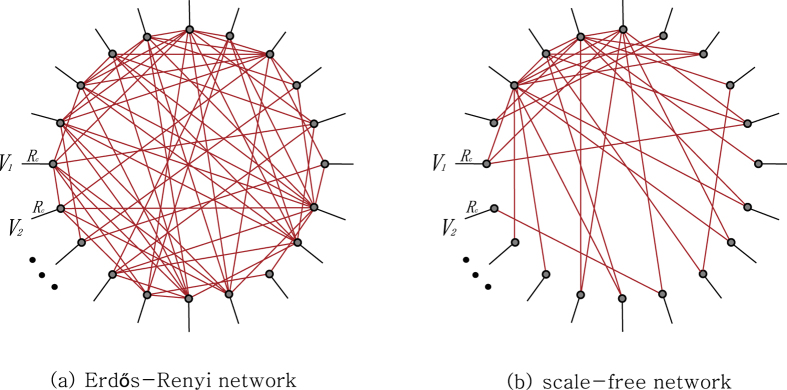
Circuits based on two types of typical random networks. Each agent connects a pair of the nodes wired to the external potentials. For a suitable visualization, the agents are denoted by red lines.

Here we will investigate the maximum power game on two types of typical random networks: Erdös-Rényi model(ER) and scale-free network. In the ER model, all nodes are equally likely connected with the same probability, say, *p*. The degree distribution of any particular node is therefore binomial. On the contrary, a scale-free network is a network whose degree distribution follows a power law, *P*(*k*) ~ *k*^−*γ*^. It has been reported many real world networks are scale-free, at least asymptotically. We use the preferential attachment method to generate scale-free networks. Figure 11 illustrates examples of the two circuits which are based on ER and scale-free networks.

**Figure 12:**
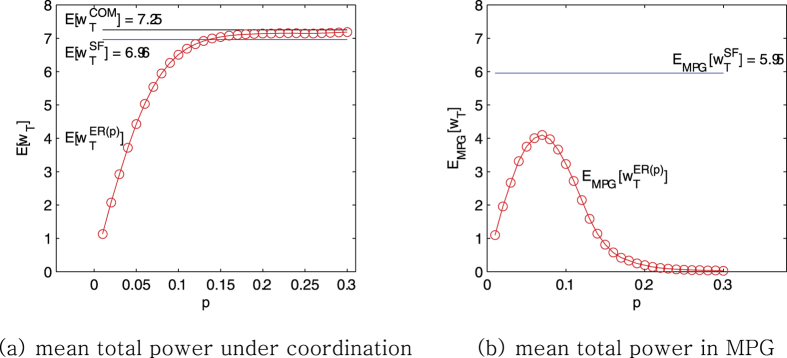
Comparisons of the mean total powers in networks according to the network topology. The comparisons are performed for two types of the networks(ER and scale-free) under two different conditions(complete coordination and the maximum power game). The mean total power for the ER network, 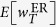 and 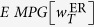 are graphed according to the connection rate *p* and are compared to the mean total power for the scale-free network 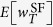 and 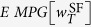, respectively. The standard deviations of 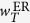 and 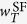 in (**a**) are around 1.88 and 1.86, respectively. The standard deviations of 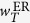 and 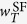 in (**b**) are around 1.43 and 1.62, respectively).

Consider ensembles of the three types of network circuits: complete, ER and scale-free networks. They are all connected to 30 external voltages *V*_1_, *V*_2_, 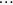, *V*_30_ with the fixed resistance *R*_*c*_ = 1. For each kind of networks, we create 1000 networks of the same nature with the external voltages following the normal distribution *N*(0, 1). Let us denote by 
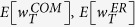
 and 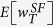 the mean of the possible maximum power induced by full cooperation of the agents in complete, ER and scale-free networks, respectively. These are not the results from the maximum power games, but from fine coordination of the resistors for comparison. As to the complete networks, the mean of the maximum producible power is 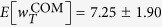. Note that this is consistent with the mean evaluated by using (16). Figure 12(a) shows that the mean total power of the ER circuits, 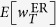, gradually increases from 0 to 7.25, as the connection probability *p* rises. The standard deviation stays around 1.88 along *p*. The mean of the maximum producible power for the scale-free networks is 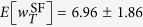.

What we are interested in is how much total power is generated against 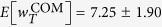, from the maximum power games. Let us denote by 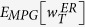 and 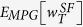 the mean total power induced by the maximum power games, of ER and scale-free networks, respectively. In Fig. 12(b), the mean of the total power from the ER model increases with *p*, reaching its maximum 4.1 at around *p* = 0.075. The standard deviation stays around 1.43 along *p*. However, raising *p* further reduces the total power and eventually makes it zero. This agrees with the fact that the maximum power game in the complete networks leads to trivial equilibrium. So if the connections are randomly made without any structure, they may cause individual competitions to harm collective performance.

On the contrary, in the scale-free networks, the mean total power that the maximum power game generates, 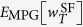 is 5.95 ± 1.62. This is decent compared to the maximum with full coordination, 6.96 ± 1.86 (and even to 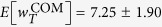). Hence, as long as the network has the scale-free structure, competition between people does not necessarily conflicts with the public good, and the maximum power game yields better collective performance.

## Discussion and Perspectives

This work shows that, in order to promote the collective power through the maximum power games, we need to have either 1) a system with more complex topology than a simple combination of parallel/serial circuits, or 2) nonhomogeneous players with various efficiency. In addition, especially with multiple inputs, the system with the scale-free structure is more advantageous than dense connections or random connections.

How a group maintains, and even prospers, with its members pursuing their own profit is a fundamental problem in economics and ecology. Conventional game theories often employ the dichotomy of cooperators/defectors to describe group dynamics. While the cooperation allows the group to thrive, one needs to explain how the cooperators survive in competition with the defectors. The maximum power game theory provides an alternative approach without explicitly introducing cooperation. It shows that the collective selfish efforts can lead to the success of the group as long as the group has proper structures.

The maximum power game provides with insight and tools to deal with collective phenomena occurring among agents who are interacting and competing for common resource. It can cover a wide range of phenomena, from a social dilemma in which the whole group loses to a more well-coordinated situation in which the pursuit of private profit promotes the collective outcomes. We believe that the maximum power game is a flexible framework to study mutual influence of competitions, distribution of resources and collective efficiency.

We assumed in this work that the environment constantly provides with fixed potential energy. The maximum power games with time-varying external voltage sources are applicable to more extensive problems. In such systems, involving “smart” agents seems to be necessary: They choose their actions based not only on the current feedback but also on the previous data. They may accumulate the potential energy using capacitors to prepare for a sudden change of the environment. Combining the game theory with the stochastic optimal control theory, the future work will drive researches for energy applications such as the optimal power flow problem and the massive power grid failure problem.

## Additional Information

**How to cite this article**: Kim, P. Maximum Power Game as a Physical and Social Extension of Classical Games. *Sci. Rep.* **7**, 43649; doi: 10.1038/srep43649 (2017).

**Publisher's note:** Springer Nature remains neutral with regard to jurisdictional claims in published maps and institutional affiliations.
